# Different clinical courses of various radiologic findings in fibromuscular dysplasia during a 7-year follow-up: A case report

**DOI:** 10.1097/MD.0000000000021108

**Published:** 2020-07-10

**Authors:** Sangil Park, Hye-Yeon Choi

**Affiliations:** Department of Neurology, Kyung Hee University College of Medicine, Kyung Hee University Hospital at Gangdong, Seoul, Republic of Korea.

**Keywords:** angiography, dissection, fibromuscular dysplasia, natural history, symptom

## Abstract

**Rationales:**

The natural history of fibromuscular dysplasia (FMD) is unclear. Furthermore, the correlation between radiologic findings and clinical significance has not been documented. Previously, the development of new vascular symptoms was reported in a small number of patients, but some of these symptoms were from other vascular causes. New arterial lesions were rarely observed during follow-up in the previous reports.

**Patient concerns:**

A 40-year-old man was admitted due to dysarthria and left-sided weakness. He had developed flank pain due to bilateral renal infarction about 10 months earlier. He had no known risk factors for atherosclerosis. Initial neurological examination revealed a mild weakness and central facial palsy on the left side.

**Diagnoses:**

Diffusion-weighted magnetic resonance imaging revealed a small acute infarction in the right insular cortex. Magnetic resonance angiography and digital subtraction angiography showed a severe stenosis with post-dilatation in the right internal carotid artery (ICA). There was a focal ectatic lesion in the left ICA. On the previous abdominal computed tomography angiography (CTA), there were arterial lesions suggestive of dissection in the bilateral renal arteries and a rod-shaped ectasia in the left common iliac artery (CIA). The pathological diagnosis was mixed-type FMD involving the intima and media.

**Interventions:**

The patient was prescribed antiplatelet agents for prevention of further ischemic events and followed up regularly.

**Outcomes:**

Seven years after the initial renal infarction, the patient developed abdominal pain radiating to the back. Abdominal CTA revealed that an aortic dissection had developed in the infrarenal aorta, which was shown as normal previously. The ectasia in the left CIA and left ICA showed no interval changes during follow-up.

**Lessons:**

We present a patient who developed spontaneous symptomatic dissection of the bilateral renal arteries, right ICA, and abdominal aorta during 7 years of follow-up, which were caused by pathologically confirmed FMD. Besides the symptomatic multifocal dissection, the patient showed an asymptomatic multifocal ectasia on cerebral and abdominal angiographies that had not changed over 7 years.

## Abstract 2

Supplemental Digital Content is available in the text

## 1 Introduction

Fibromuscular dysplasia (FMD) is an idiopathic arterial disease that is non-inflammatory and non-atherosclerotic.^[1]^ It can affect all layers of the artery wall and can be found in all territories.^[1]^ The natural history of FMD is unclear, but it usually shows a low recurrence rate.^[2–4]^ We present a patient who developed spontaneous symptomatic dissection of the bilateral renal arteries, right internal carotid artery (ICA), and abdominal aorta during 7 years of follow-up, which were caused by pathologically confirmed FMD. Besides the symptomatic multifocal dissection, the patient showed an asymptomatic multifocal ectasia on cerebral and abdominal angiographies that had not changed over 7 years.

## 2 Case report

The ethical approval and informed consent were waived by the Institutional Review Board (IRB) of Kyung Hee University Hospital at Gangdong due to the retrospective nature of the analysis and lack of private information, following the enforcement rules (article 13 and article 33 of Bioethics and Biosafety Act) and article 9 of the KHNMC SOP (IRB No. KHNMC 2019-11-004).

A 40-year-old man was admitted to the neurology department due to dysarthria and left-sided weakness. He had developed bilateral renal infarction, which presented with flank pain about 10 months earlier, and occurred in each side within a 1-month interval. Since then, the patient had been taking an oral vitamin K antagonist. He had no known risk factors for atherosclerosis, such as hypertension, diabetes mellitus, or dyslipidemia, and no familial history of any medical diseases. He had never been a smoker. His blood pressure and heart rate were normal. Initial neurological examination revealed a mild weakness and central facial palsy on the left side. Diffusion-weighted magnetic resonance imaging (MRI) revealed a small acute infarction in the right insular cortex. MR angiography and digital subtraction angiography (DSA) showed a severe stenosis with post-dilatation in the right ICA. A dissecting aneurysm was noted on the follow-up DSA that was performed 1 month later. After reviewing previous abdominal computed tomography angiography (CTA) that was suggestive of dissection in the bilateral renal arteries, a temporal artery biopsy was performed. The biopsy showed a focal disruption of the internal elastic lamina with a disorganized tunica media and fibrointimal proliferation (Supplemental Figure) that was compatible with mixed-type FMD involving the intima and media. The patient was prescribed antiplatelet agents and followed up regularly. Seven years after the initial renal infarction, the patient visited the emergency department due to periumbilical pain radiating to the back. Abdominal CTA showed an abdominal aortic dissection of the infrarenal aorta. The patients blood pressure was 157/96 mm Hg. He was managed conservatively and was discharged after clinical stabilization.

### 2.1 Radiologic findings

#### 2.1.1 Symptomatic vascular lesions

**Figure 1:**
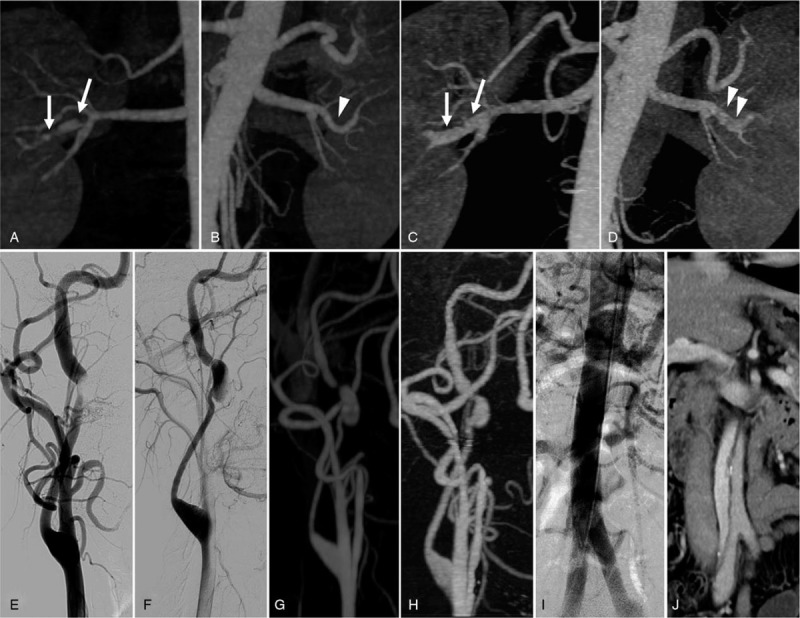
Radiologic findings of symptomatic lesions during the follow-up period. Initially observed multifocal stenosis with post-dilatation in the right renal artery (A, arrows) and normal left renal artery (B, arrowheads). On follow-up renal computed tomography angiography (CTA), the right renal artery showed dilatation (C, arrows), and the left renal artery showed luminal irregularity with slight dilatation (D, arrowheads). Initial cerebral angiography showed a tapered stenosis with abrupt post-dilatation in the mid-cervical internal carotid artery (E) and a dissecting aneurysm was observed on follow-up (F). There was no further interval change observed on follow-up (G, H). Newly developed dissection was noted in the infrarenal abdominal aorta (J), which was previously normal (I) on abdominal CTA.

First abdominal CTA after right renal infarction showed several stenotic lesions with post-dilatation in the right renal artery (Fig. 1A), while the left renal artery was normal (Fig. 1B). The following abdominal CTA after left renal infarction showed dilatation in the right renal artery (Fig. 1C) and newly developed luminal irregularity with dilatation in the left renal artery (Fig. 1D). DSA performed after the diagnosis of stroke showed a steeply tapered stenosis in the mid-portion of the cervical ICA with abrupt dilatation in the post-stenotic area, which was suggestive of arterial dissection (Fig. 1E). A dissecting aneurysm was noted on the follow-up DSA that was performed 1 month later (Fig. 1F). On follow-up neck CTA performed 5 years later, there was no significant interval change in the right ICA dissecting aneurysm (Fig. 1G). There was again no change in the right ICA on CTA over 7 years, (Fig. 1H); however, an abdominal aortic dissection had developed in the infrarenal aorta (Fig. 1J), which was shown as normal in previous CTA (Fig. 1I).

#### 2.1.2 Asymptomatic vascular lesions

**Figure 2:**
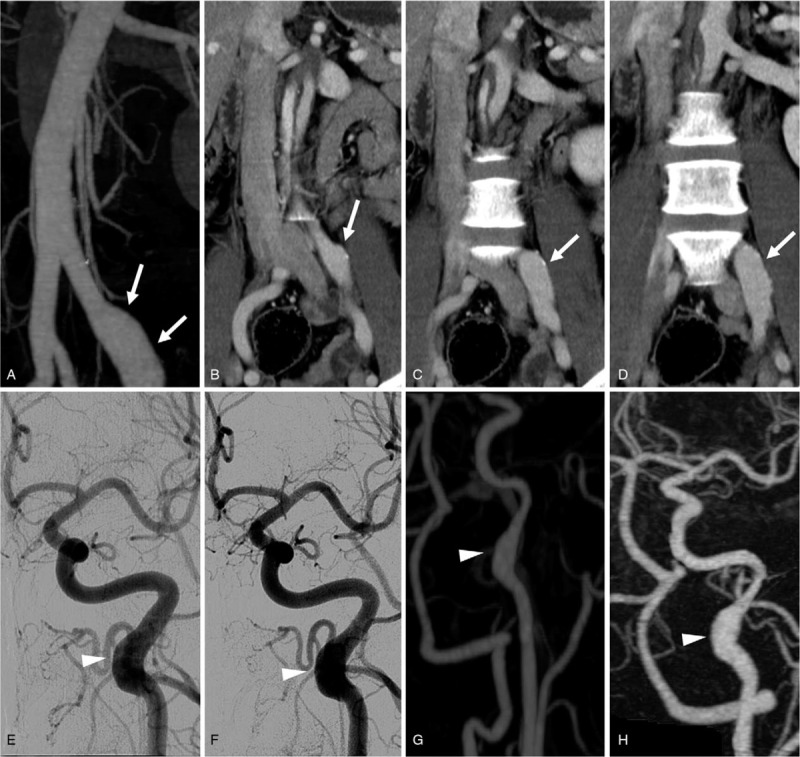
Radiologic findings of asymptomatic lesions during the follow-up period. There was a rod-shaped dilated lesion (arrows) in the left common iliac artery (A) with no interval changes observed over 7 years (B-D). Another rod-shaped dilated lesion (arrowheads) was observed in the left internal carotid artery (E) with no interval changes during follow-up (F-H).

There was a rod-shaped ectatic lesion in the left common iliac artery on the first abdominal CTA (Fig. 2A), and no significant interval change was observed during follow-up evaluations (Fig. 2B-D). There was a dilated area in the C2 portion of the left ICA without an interval change during the follow-up (Fig. 2E-H).

## 3 Discussion

The patient in the present case showed mixed radiologic findings in different arterial beds, including stenosis with post-dilatation, irregularity with slight dilatation, dissecting aneurysm, and rod-shaped ectasia. Four lesions had related clinical symptoms and the other 2 were asymptomatic. Although there are no concrete radiologic diagnostic criteria for FMD, a string of beads appearance has been suggested as a typical radiologic finding.^[5,6]^ Several other findings have been reported as supportive of FMD, such as tubular or localized unifocal stenosis, a web-like defect, and various forms of vascular dilatation, from corrugated diverticulum-like outpouching with noncircumferential narrowing to a true aneurysm.^[5,6]^ A correlation between radiologic findings and clinical significance has not been documented. In the present case, the dissection and stenosis were symptomatic, and the rod-shaped ectasia was asymptomatic.

The natural course of FMD is not well known. The development of new vascular symptoms was reported in a small number of patients (2%–5.9%) but some of these symptoms were from other vascular causes.^[3,4]^ New arterial lesions were rarely observed during follow up.^[2–4]^ In the present case, a newly developed aortic dissection was noted at a region that previously showed no abnormality. The irregularly dilatated lesion in the left renal artery was also normal on previous CTA. Both lesions were symptomatic. On the contrary, the 2 ectatic lesions showed no interval radiologic changes and did not induce any clinical symptoms during the 7 years of follow-up.

The present case suggests possible radiologic and symptomatic characteristics of the natural history of FMD. Asymptomatic ectatic lesions are unlikely to show morphologic or symptomatic interval changes. New symptomatic lesions could occur at previously normal arterial regions. Further large observational studies are necessary to provide a more detailed natural history of FMD.

## Author contributions

**Conceptualization:** Hye-Yeon Choi.

**Data curation:** Sangil Park.

**Writing – original draft:** Sangil Park.

**Writing – review & editing:** Hye-Yeon Choi.

## Supplementary Material

Supplemental Digital Content
