# Supplementary material for: Different clinical courses of various radiologic findings in fibromuscular dysplasia during a 7-year follow-up: A case report
Source: Medicine (Baltimore). 2020 Jul 10;99(28):e21108. doi: 10.1097/MD.0000000000021108 (PMC7360207; doi:10.1097/MD.0000000000021108)

Supplemental Figure. Temporal artery biopsy.
The temporal artery biopsy revealed a focal disruption of the internal elastic lamina with a disorganized tunica media. The intimal (arrows) and medial layers (arrowheads) showed abnormal proliferation.

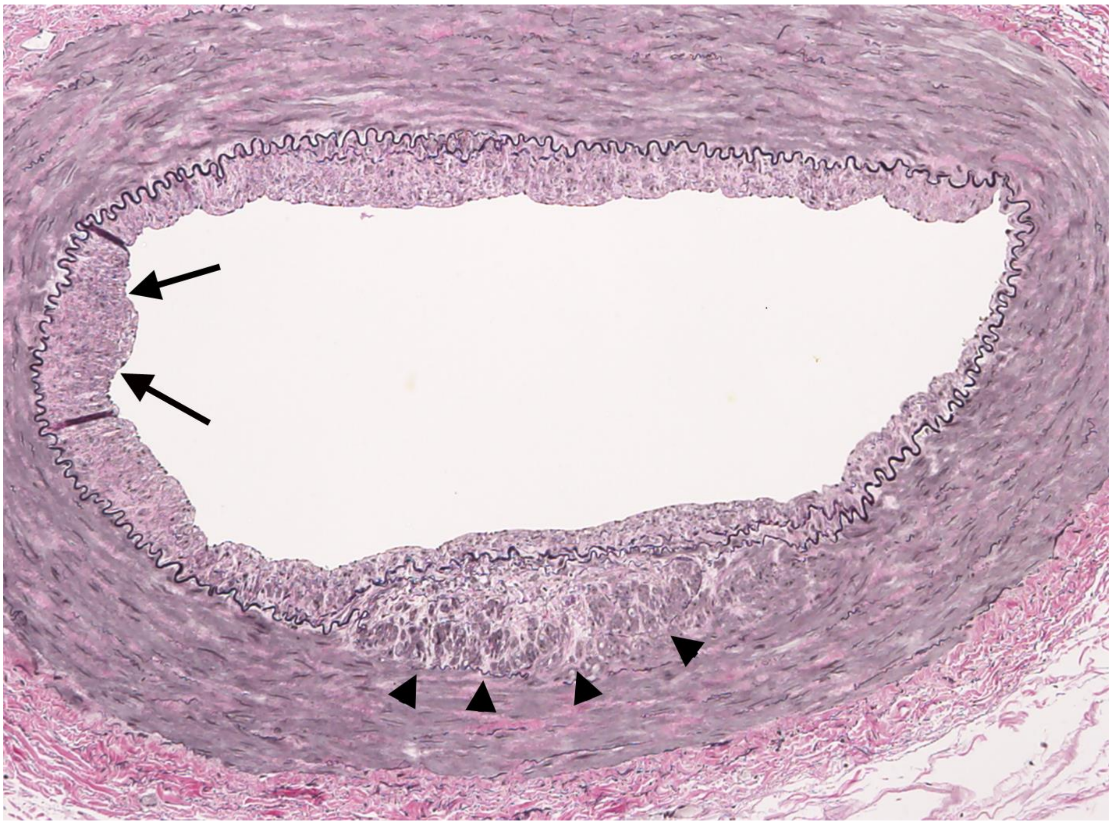

Supplement: Supplemental Digital Content [file medi-99-e21108-s001.pdf]
